# Organ Identity Outweighs Geographic Origin in Shaping the Metabolome of *Tetrastigma hemsleyanum*

**DOI:** 10.3390/metabo16090619

**Published:** 2026-08-27

**Authors:** Mingli Ye, Yukai Ba, Zhengrui Chen, Boya Liu, Xin Li, Xiaoya Yu, Yonggang Zhao, Ban Cao, Chao Lei, Yu Liu

**Affiliations:** 1 College of Biological and Environmental Engineering, Zhejiang Shuren University, Hangzhou 310015, China; 2 State Key Laboratory of Environmental Chemistry and Ecotoxicology, Research Center for Eco-Environmental Sciences, Chinese Academy of Sciences, Beijing 100085, China; 3 Zhejiang Institute of Geosciences, Hangzhou 310000, China; 4 College of Life Sciences, Zhejiang University, Hangzhou 310058, China

**Keywords:** *Tetrastigma hemsleyanum*, untargeted metabolomics, organ specificity, metabolic division of labour, annotation confidence, geographic variation, random forest

## Abstract

**Background/Objectives:** The whole plant of *Tetrastigma hemsleyanum* is used medicinally, but the tuberous root is by far the most commonly used part, and the way in which its metabolites are partitioned among organs and among geographic origins underpins both the rational choice of the medicinal part and the evaluation of herb quality. **Methods:** Here, untargeted metabolomics based on ultra-high-performance liquid chromatography coupled to high-resolution Orbitrap mass spectrometry was used to compare roots, stems and leaves collected from nine regions of southern China (81 samples) with tuberous roots collected from 17 sites in seven provinces (51 samples). Annotations were graded according to the Metabolomics Standards Initiative (MSI), curated with explicit plausibility rules, and every conclusion was re-tested across five nested annotation subsets. **Results:** Organ identity was the dominant source of metabolic variation: the three organs were completely separable (random forest out-of-bag accuracy 100%), the organ effect was about four times larger than that of sampling region (PERMANOVA pseudo-F 16.7 versus 4.1), and this contrast was essentially unchanged from the complete set of 615 annotations down to the most stringent subset of 19 flavonoids and phenolic acids. The organ-level pattern was chemically coherent: amino acids and lipids were relatively enriched in the tuberous root, soluble sugars and phenolic acids in the stem, and flavonoids and alkaloids in the leaf, matching the contrasting roles of a storage, a transport and a photosynthetic organ. Geographic differences among tuberous roots were, by contrast, weak and largely local: although provinces could be separated with 88.9% out-of-bag accuracy, accuracy fell to 42.2% when an entire, previously unseen collection site was held out (chance level 20%), and to 52.9% for a coarse macro-geographic zone (chance level 25%), whereas holding out an entire sampling region left organ classification unaffected (100%). **Conclusions:** The present data therefore document a strong, generalisable and chemically interpretable organ division of labour, but do not support the use of this metabolome for origin authentication. Because most annotations remain at MSI Level 3, individual compounds are reported as putative throughout, and all conclusions rest on multivariate and class-level evidence.

## 1. Introduction

*Tetrastigma hemsleyanum* Diels et Gilg is a perennial climbing vine of the family Vitaceae and an important traditional and ethnomedicinal herb in southern China, valued for clearing heat, removing toxins and promoting blood circulation. Modern pharmacology has confirmed antitumor, anti-inflammatory, antioxidant and immunomodulatory activities, and flavonoids are regarded as a major material basis of its efficacy [1,2,3]. Although the whole plant can be used medicinally, both traditional and current applications rely mainly on the tuberous root, while stems and leaves are often regarded as by-products or discarded [1]. Reports of antitumor and anti-inflammatory effects of *T. hemsleyanum* flavonoids and polysaccharides [4,5,6,7] further underscore the importance of clarifying where its active constituents accumulate.

Organ-specific division of secondary metabolism is a general phenomenon in plants: within one individual, roots, stems and leaves differ markedly in the biosynthesis, transport and accumulation of metabolites owing to their distinct functions (storage, support, photosynthesis), and this organ specificity directly determines the choice of medicinal part and the quality of the herb [8,9]. Flavonoids and phenolic acids are synthesised through the phenylpropanoid–flavonoid pathway and are finely regulated in a tissue- and environment-dependent manner [10,11,12,13,14], and these constituents display broad antioxidant, anti-inflammatory and antitumor activities [15,16,17,18,19,20]. For *T. hemsleyanum*, however, studies have mostly focused on the tuberous root itself or on its geographic origin [21,22,23], whereas a systematic comparison of roots, stems and leaves is still lacking, and whether the aerial parts are rich in distinctive active constituents that could be valorized remains unexplored. In parallel, geographic origin (authenticity) is another important dimension of quality, but existing origin studies have reached inconsistent conclusions [21,24]. Which of the two—organ identity or growing environment—dominates the metabolome is not a foregone conclusion, and the balance differs among species and among compound classes. In a cosmopolitan weed, geographic origin was found to be the single strongest predictor of metabolic phenotype, outweighing tissue and even genetic background [25]. In grapevine, the berry metabolome is so plastic with respect to vineyard site that the site of production can exceed cultivar in its influence on particular metabolite families, although the resulting chemical signatures are subtle and require supervised modelling to be resolved [26,27]. Conversely, in species that develop strongly differentiated storage organs, the organ effect is expected to prevail, because storage, supporting and photosynthetic tissues differ fundamentally in the pathways they express and in the metabolite pools they maintain [8,28]. The relative magnitude of the two effects is therefore an empirical question that has to be settled quantitatively for each species, and it has not been settled for *T. hemsleyanum*.

Untargeted metabolomics based on high-resolution mass spectrometry enables unbiased comparison of metabolic profiles and is widely used for organ comparison, origin authentication and quality evaluation of medicinal plants [29,30,31,32]. However, software-based database matching is prone to false-positive annotations, especially when the matrix is complex and MS/MS coverage is limited, so that many annotations remain at the MS1/database level and are of limited reliability [30,33]. Explicitly reporting the confidence level of each identification, and basing the main conclusions on robust multivariate and class-level results, is therefore essential when interpreting such data [34].

Against this background, *T. hemsleyanum* was examined here by UHPLC-Orbitrap MS-based untargeted metabolomics with MSI confidence grading, with four objectives: (i) to describe the metabolome of roots, stems and leaves collected from a common set of regions and to interpret the resulting pattern in terms of the physiological roles of the three organs; (ii) to quantify, within one data set, the relative contribution of organ identity and of geographic region to metabolic variation; (iii) to establish how far any geographic signal detected in the tuberous root generalises beyond the particular sites that were sampled; and (iv) to determine how much of the outcome depends on the quality of the annotation layer on which the chemometric analysis operates. The study is intended as a quantitative and mechanistically interpreted description of the *T. hemsleyanum* metabolome rather than as the basis of an authentication system.

## 2. Materials and Methods

### 2.1. Plant Material and Sampling

Whole-plant samples for the organ comparison were collected in March 2025 from nine regions in southern China (Nanping, Fujian; Hangzhou and Jinhua, Zhejiang; Baise, Guangxi; Enshi, Hubei; southern and northern Yongzhou, Hunan; Ganzhou, Jiangxi; and Wanyuan, Sichuan). All were cultivated green-vine plants from managed production sites, not wild-collected populations, and each individual was separated into root, stem and leaf, with three biological replicates per organ per region, giving 81 samples. Samples were collected as *T. hemsleyanum* based on field morphology, including a slender climbing habit, trifoliolate leaves, green stems and swollen tuberous roots. A representative plant photographed during sampling, together with the separated leaves, stems and tuberous roots, is shown in Figure S4. A formal voucher specimen was not retained.

Tuberous-root samples for the origin comparison were collected in April 2025 from 17 sites across seven provinces (Zhejiang, Fujian, Guizhou, Jiangxi, Guangdong, Guangxi and Hunan), all cultivated plants from managed production sites, with three biological replicates per site, giving 51 samples. In both sample sets, the underground organ analysed was the swollen tuberous root, which constitutes the medicinal material; fibrous roots were discarded. “Root” in the organ comparison and “tuberous root” in the origin comparison therefore refer to the same organ. The two data sets differ in sampling design and analytical batch. Three geographic levels are used throughout: site, the individual collection locality; province, the administrative unit; and macro-geographic zone, a coarse grouping based on broad climatic and physiographic setting (Table 1). All samples were freeze-dried, ground, passed through a 100-mesh sieve and stored at −80 °C.

### 2.2. Sample Preparation and Instrumental Analysis

HPLC-grade methanol and acetonitrile were purchased from Fisher Scientific (Waltham, MA, USA), HPLC-grade formic acid was purchased from Sigma-Aldrich (St. Louis, MO, USA), and ultrapure water was produced using a Milli-Q Advantage A10 system (Merck Millipore, Burlington, MA, USA). A 1 g portion of lyophilized powder was extracted with 1.2 mL methanol/water (70:30, *v*/*v*; vortexed for 6 s every 30 min, six times; 3 h total), centrifuged (10,000 r/min, 10 min) and filtered through a 0.22 µm membrane. Quality-control (QC) samples were prepared by pooling equal aliquots of all extracts.

Chromatographic separation was performed using either a Vanquish UHPLC system (Thermo Fisher Scientific, Waltham, MA, USA) or an LC3600N NanoLC system (Anhui Wanyi Science and Technology Co., Ltd., Hefei, China). Both systems used the same Acclaim RSLC 120 C18 column (2.2 µm, 2.1 mm × 100 mm; Thermo Fisher Scientific, Waltham, MA, USA) under identical chromatographic conditions. The mobile phases were 0.1% formic acid in water (A) and acetonitrile (B), with a 5–30–95–95–5% B gradient over 12.1 min at 0.35 mL/min, a column temperature of 30 °C and a 5 µL injection. Detection used an Orbitrap Exploris 240 mass spectrometer with a heated electrospray ionisation source (Thermo Fisher Scientific, Waltham, MA, USA). Positive- and negative-mode spray voltages were 3500 and 3000 V, respectively; sheath, auxiliary and sweep gas were 40, 10 and 0 arbitrary units; and the ion transfer tube and vaporiser temperatures were 320 and 350 °C. Full-scan spectra were acquired at a resolution of 120,000 over *m*/*z* 100–1500. The six most intense precursor ions above 5 × 10^4^ counts were selected for stepped HCD at 30, 50 and 70 eV, with MS2 acquired at a resolution of 15,000. EASY-IC was used for scan-to-scan internal mass calibration. QC injections were inserted after every six samples.

### 2.3. Data Processing and Confidence Grading

Raw data were processed in Compound Discoverer 3.3 (Thermo Fisher Scientific; positive and negative modes separately; mass error ≤ 5 ppm, S/N ≥ 3) against mzCloud (https://www.mzcloud.org/; accessed on 26 August 2026), the mzVault and MassList libraries supplied with Compound Discoverer 3.3, and ChemSpider (https://www.chemspider.com/; accessed on 26 August 2026). Compounds with a peak-area rating below 7 were discarded, leaving 615 annotated compounds (153 in positive and 462 in negative mode). Because only a minority had MS/MS library matches, each compound was graded following the MSI: Level 2 for a strong mzCloud MS/MS match and Level 3 for weak MS/MS similarity or MS1/database matches only. Annotations were then curated using three reproducible rules: (i) database placeholder identifiers and tentative “similar to” entries were removed; (ii) formulas containing elements other than C, H, N, O, P and S were removed; and (iii) annotations corresponding by name to synthetic drugs, agrochemicals, industrial chemicals or animal-specific metabolites were removed. These rules removed 6, 167 and 69 entries, respectively, and retained 373 features, of which 75 could be assigned to a chemical class. Analyses were repeated on five nested subsets: S1, all 615 annotations; S2, 373 plausible plant metabolites; S3, 75 chemically assignable metabolites; S4, 38 assignable secondary metabolites; and S5, 19 flavonoids and phenolic acids. The curated set of 565 features used in the original analysis lies between S1 and S2. Individual metabolites are reported as putative unless supported at MSI Level 2.

For direct inspection of identification evidence, positive- and negative-mode Thermo RAW files were read with AlphaRaw 0.6.1. Extracted-ion chromatograms were generated with a ±5 ppm window. A target was considered to have DDA coverage only when the isolated precursor was within ±5 ppm of the queried *m*/*z*. Six isobaric ion formulas were queried in each mode: the kaempferol/luteolin aglycone and ions corresponding by exact mass to afzelin, astragalin/kaempferol-O-hexoside, a hexoside-rhamnoside, a dihexoside and a hexoside-dirhamnoside. Screening covered all 132 study samples, nine regional QC files, six pooled-QC files and six solvent blanks in each mode. An expanded product-ion search required an ion within ±5 ppm of the kaempferol/luteolin-type aglycone at an intensity of at least 1% of the spectrum base peak; common glycoside neutral losses were evaluated within 20 ppm. Exact-mass chromatographic signals were not treated as compound identifications without matching DDA evidence.

### 2.4. Statistical Analysis

Peak areas were normalised to total peak area, log2-transformed and scaled to unit variance for multivariate analyses. Principal component analysis (PCA) was used for unsupervised comparison. Permutational multivariate analysis of variance (PERMANOVA, Euclidean distance, 999 permutations) was used to quantify the contributions of organ and sampling region within the organ data set, and of site, province and macro-geographic zone within the origin data set.

Effect sizes were compared using the degrees-of-freedom-adjusted pseudo-F rather than R2, because R2 increases mechanically with the number of groups and is not directly comparable between factors with different numbers of levels. Random forests with 1000 trees were used for supervised classification [35]. Performance was assessed by (i) out-of-bag estimation and repeated stratified cross-validation; (ii) a permutation test in which class labels were reassigned 99 times; and (iii) leave-one-site-out validation, or leave-one-region-out validation for the organ data set. In the third scheme, all three replicates from one collection site were held out together. Only classes represented by at least two sites could be evaluated, which restricted the province-level analysis to five provinces (15 sites, 45 samples).

Differential metabolites were screened using |log2 fold change| > 1 and Student’s *t*-test *p* < 0.05. Organ enrichment was defined as a group mean more than two-fold higher than the highest of the other two organs. Class relative content was calculated as the summed normalised intensity per class across organs, and KEGG over-representation analysis was performed for differential metabolites [36]. Analyses were implemented in Python 3 (Python Software Foundation; https://www.python.org/; accessed on 26 August 2026) using scikit-learn (https://scikit-learn.org/), SciPy (https://scipy.org/) and statsmodels (https://www.statsmodels.org/; all accessed on 26 August 2026). The curation rules, feature subsets and analysis scripts were provided to support numerical reproducibility.

## 3. Results

### 3.1. Organ Identity Strongly Structures the Metabolome

**Figure 1:**
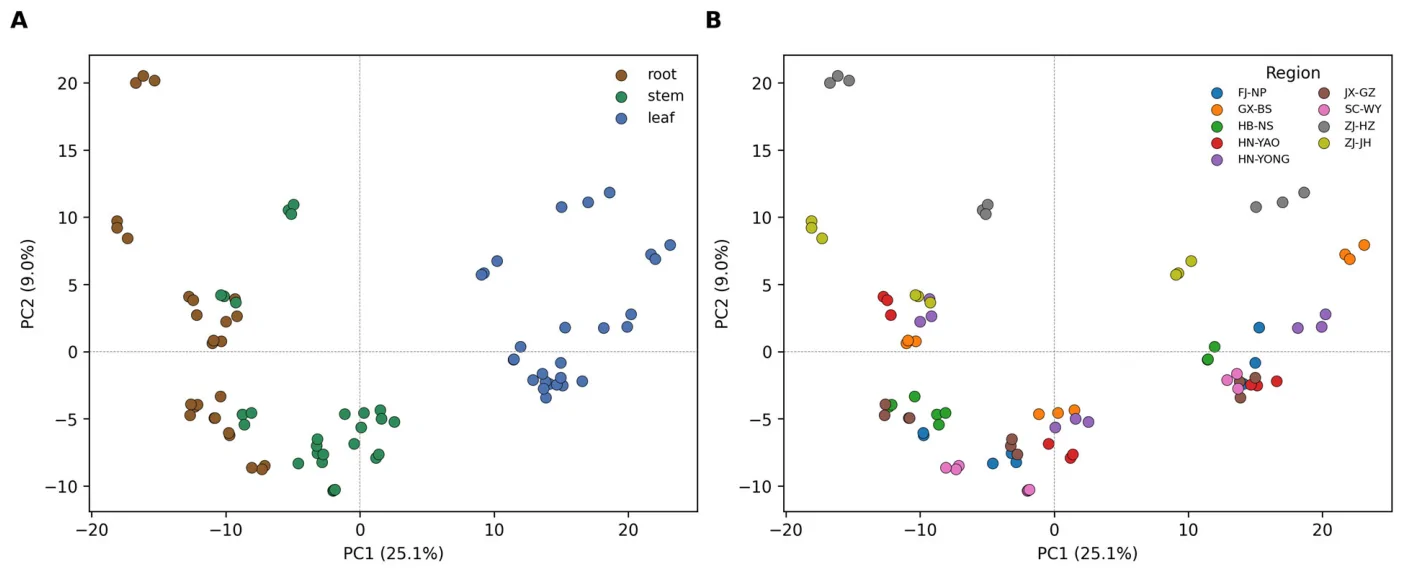
PCA score plots of metabolites in the roots, stems and leaves of *T. hemsleyanum*. (**A**) Coloured by organ; (**B**) coloured by sampling region.

PCA of the 81 root, stem and leaf samples from nine regions (Figure 1) separated the three organs clearly. Leaf samples were displaced from root and stem along PC1 (25.1%), and root and stem were also distinguished. The same samples showed no clustering when coloured by region (Figure 1B). Random forest assigned every sample to the correct organ (out-of-bag accuracy 100%), confirming that the root, stem and leaf metabolomes were separable.

### 3.2. Organ Effect Far Exceeds Geographic Region

PERMANOVA showed that both organ and sampling region contributed significantly to metabolic variation (*p* = 0.001 in both cases), but their effect sizes differed by about fourfold (Table 2): pseudo-F was 16.7 for organ and 4.1 for region. R^2^ was similar for the two factors only because region comprised nine groups against three for organ; once the numbers of groups are accounted for, the structuring effect of organ is about four times that of region, and organ alone defined the primary axis of the PCA. The confusion matrix of the random-forest organ classification is given in Figure S1. Organ identity is therefore the primary factor structuring the *T. hemsleyanum* metabolome.

**Table 2:** Contributions of organ identity and of geographic origin to metabolic variation in *T. hemsleyanum*, tested by PERMANOVA.

Data Set	Factor	Definition of the Factor	Levels	df	Pseudo-F	*p*
Organ set (81 samples)	Organ	The plant part analysed: tuberous root, stem or leaf of the same individual	3	2, 78	16.7	0.001
Organ set (81 samples)	Sampling region	The nine localities from which whole plants were collected (Table 1)	9	8, 72	4.1	0.001
Origin set (51 samples)	Site of origin	The 17 localities from which tuberous roots were collected (Table 1)	17	16, 34	7.8	0.001

Table note: PERMANOVA was computed on Euclidean distances of total-area-normalised, log2-transformed and unit-variance-scaled data with 999 permutations. Effect sizes of factors with different numbers of levels are compared using the degrees-of-freedom-adjusted pseudo-F rather than R^2^, because R^2^ increases mechanically with the number of groups. The two data sets were collected and analysed as separate batches; pseudo-F values are therefore compared within, not between, data sets. Province- and zone-level results are given in Table 3.

**Table 3:** Sensitivity of the principal results to the stringency of annotation curation.

Annotation Subset	*n*	PC1, Organ (%)	PC1, Origin (%)	F Organ	F Region	F Province	F Zone	RF Organ OOB (%)
S1 All annotations	615	24.8	26.6	16.5	4.1	4.0	3.6	100
S2 Plausible plant metabolites	373	25.8	29.6	17.8	4.0	4.3	3.6	100
S3 Chemically assignable	75	29.7	40.4	17.9	4.2	4.2	5.0	100
S4 Assignable secondary metabolites	38	27.3	30.1	16.4	3.5	4.0	4.5	97.5
S5 Flavonoids and phenolic acids	19	30.1	47.8	16.6	4.2	3.8	4.7	100

Table note: F columns report PERMANOVA pseudo-F. Subsets are nested (S1 > S2 > S3 > S4 > S5) and are defined in Section 2.3. All PERMANOVA tests were significant at *p* = 0.001 (999 permutations). “Region” refers to the nine localities of the organ data set; “province” and “zone” refer to the administrative and macro-geographic groupings of the 17 origin sites. RF, random forest; OOB, out-of-bag.

### 3.3. Differential Metabolites Among Organs

Differential metabolites among organs were screened by |log2 FC| > 1 and *p* < 0.05 (Figure S2A–C): 168 between stem and root (123 higher in stem, 45 lower), 317 between leaf and root (205 higher in leaf, 112 lower) and 261 between leaf and stem (149 higher in leaf, 112 lower). Comparisons involving leaf yielded the most differences, consistent with the position of leaf in the PCA. Defining organ-enriched metabolites as more than two-fold higher than in either of the other two organs gave 138 leaf-enriched, 42 stem-enriched and 34 root-enriched metabolites (Figure S2D).

### 3.4. Metabolic Class Specialisation Among Organs

**Figure 2:**
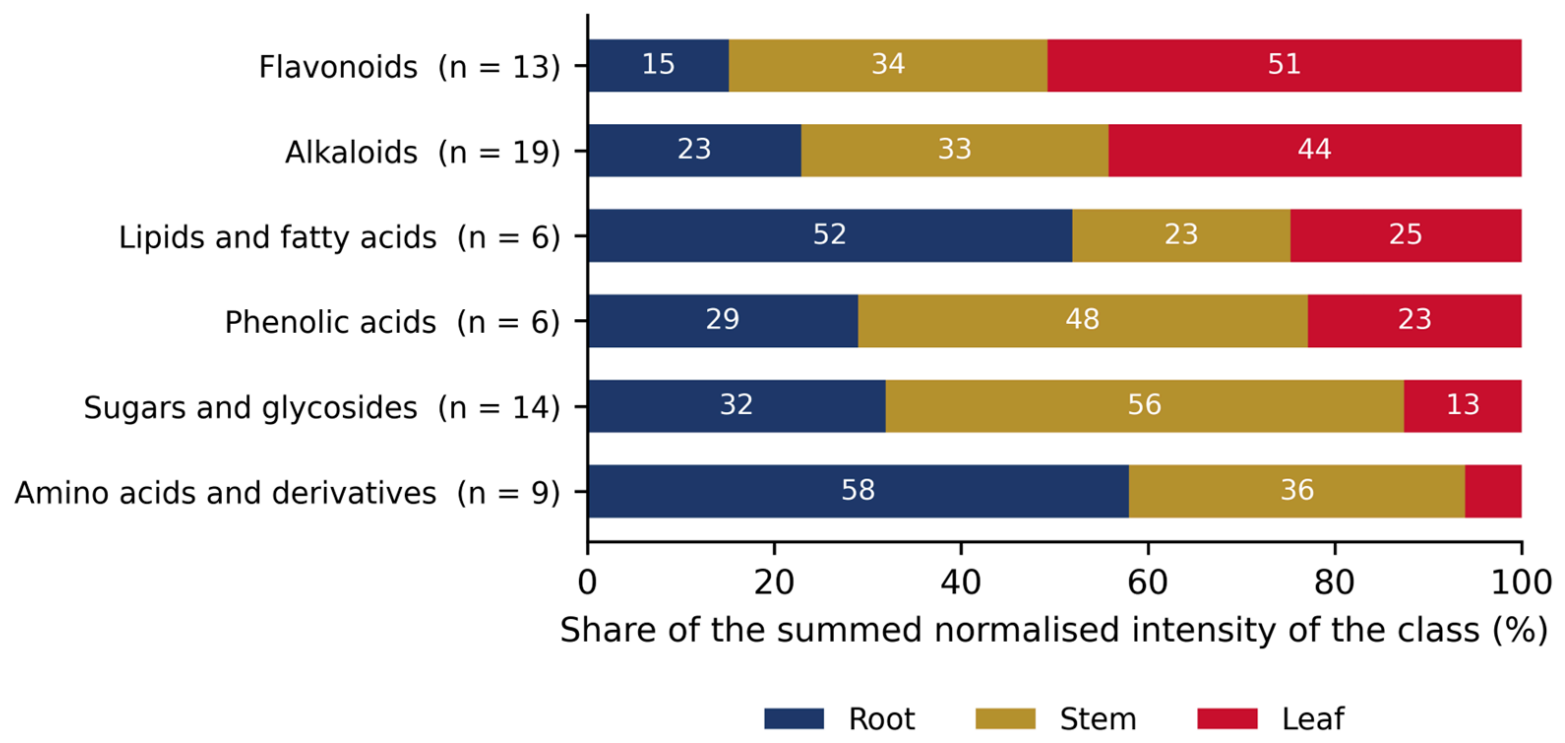
Distribution of the major metabolite classes among the roots, stems and leaves of *T. hemsleyanum*. Bars show, for each class, the percentage of the summed normalised intensity that is contributed by each of the three organs; *n* is the number of compounds assigned to the class. Only the six classes represented by at least five compounds in the curated, chemically assignable subset are shown; all class assignments are putative (MSI Level 3).

The relative distribution of each metabolite class among the three organs was computed on the chemically assignable subset, restricted to the six classes represented by at least five compounds (Figure 2). The result is a clear division of labour. The tuberous root carried the largest share of the amino acids and derivatives (58% of the summed normalised intensity of that class) and of the lipids and fatty acids (52%). The stem carried the largest share of the soluble sugars and glycosides (56%) and of the phenolic acids (48%). The leaf carried the largest share of the flavonoids (51%) and of the compounds annotated as alkaloids (44%), and the smallest share of the amino acids (6%). Because each value aggregates 6 to 19 compounds, this comparison does not depend on the identity of any individual annotation; the class assignments themselves are nevertheless putative, and classes represented by fewer than five compounds were excluded for this reason.

### 3.5. Putative Organ-Characteristic Metabolites

**Figure 3:**
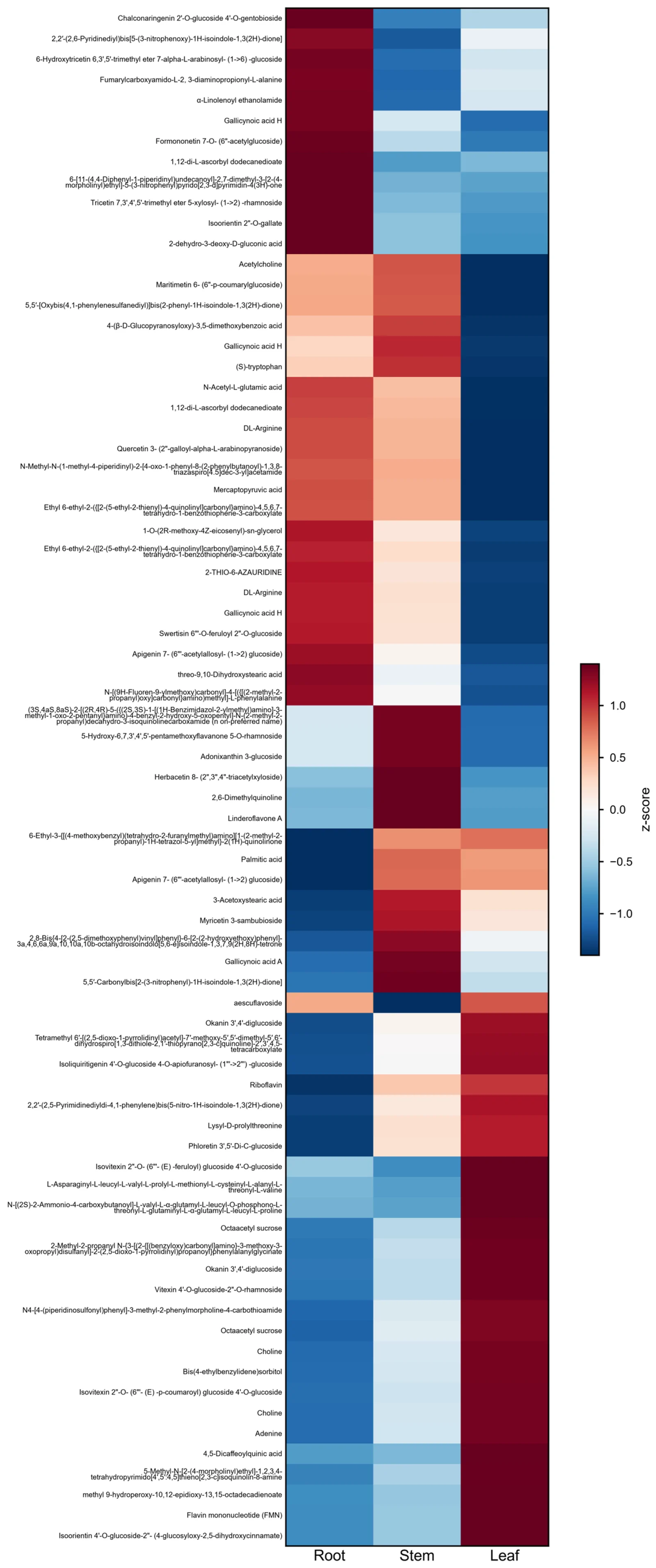
Hierarchical clustering heat map of the 75 chemically assignable metabolites across the roots, stems and leaves of *T. hemsleyanum*, shown as organ-mean z-scores. All compound identities are putative (MSI Level 3) and are used only as supporting evidence.

Hierarchical clustering of the assignable subset by organ mean (Figure 3) showed the metabolites relatively enriched in each organ. Leaves were relatively enriched in a caffeoylquinic acid annotated as 4,5-dicaffeoylquinic acid, in flavone C-glycoside conjugates of the isovitexin and isoorientin series and in a range of other glycosides; tuberous roots were relatively enriched in nitrogen-containing compounds such as an annotated N-acetylglutamic acid and in some phenolic acids and lipids; stems were intermediate. Because none of these annotations reached MSI Level 2, they are reported as putative and are used only as supporting evidence; the identification evidence available for the representative compounds is summarised in Table 4, and no conclusion in this study depends on the identity of an individual compound.

### 3.6. Regional Differences and Discriminability of Tuberous Roots Among Origins

**Figure 4:**
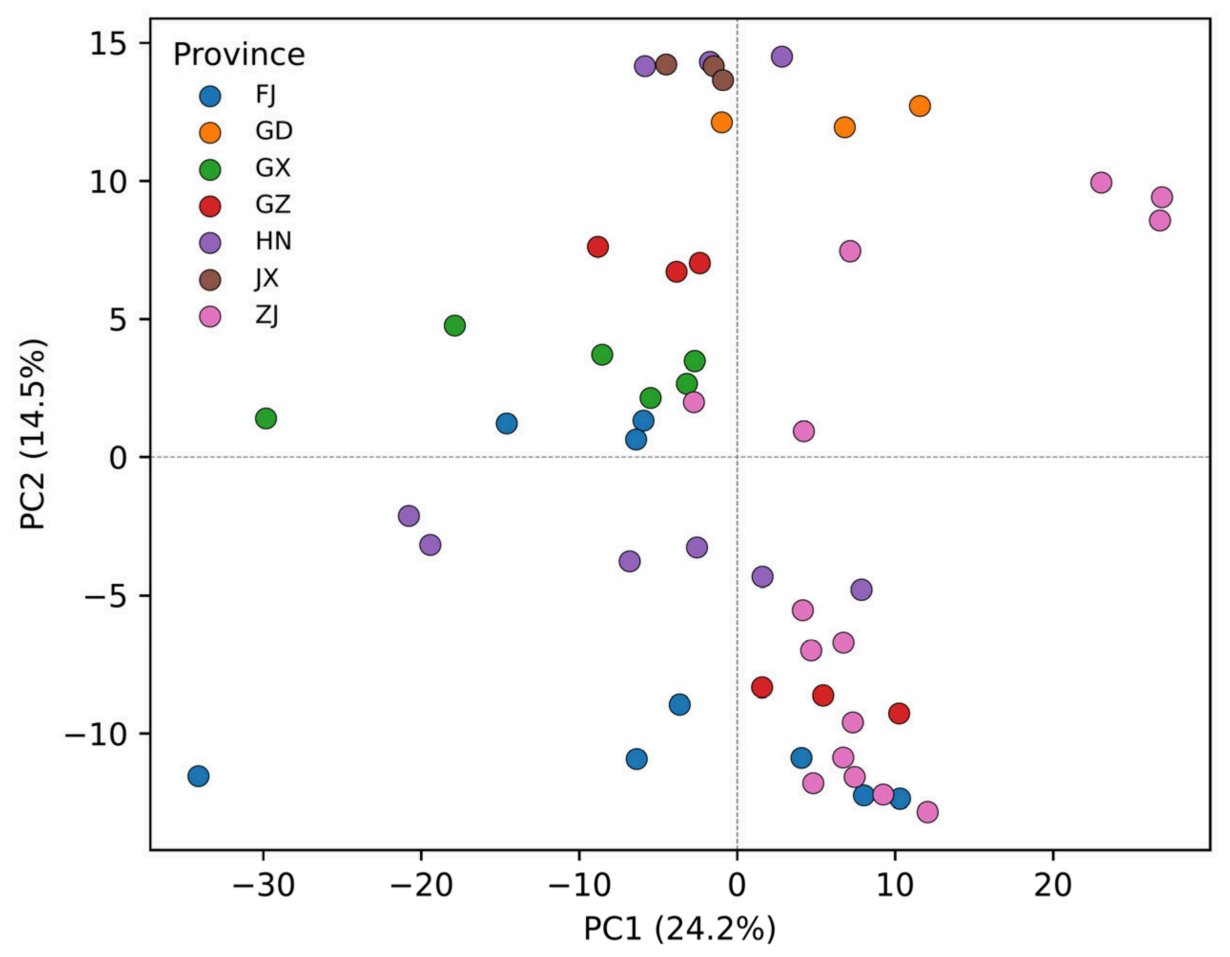
PCA score plot of the metabolomes of *T. hemsleyanum* tuberous roots from 17 sites, coloured by province. Out-of-bag and leave-one-site-out classification accuracies for the same data set are given in the text and in Figure 5C.

**Figure 5:**
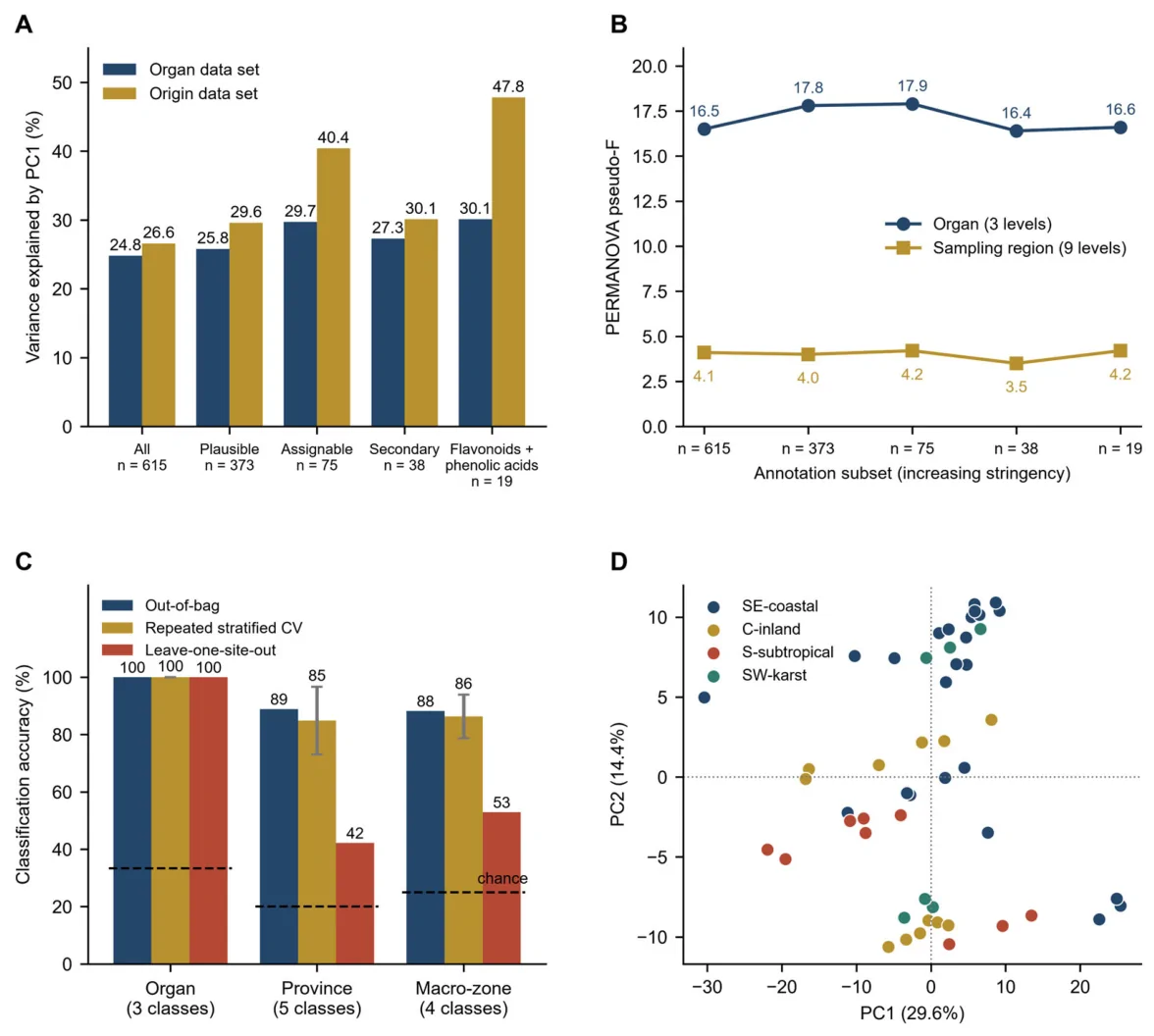
Sensitivity of the metabolome-based results to annotation stringency and to the definition of geographic units. (**A**) Variance explained by PC1 in the organ and origin data sets for five nested annotation subsets of increasing stringency (S1–S5, Section 2.3). Filtering produced a larger PC1 change in the origin data set than in the organ data set. (**B**) PERMANOVA pseudo-F for organ and sampling region across the same subsets. (**C**) Random-forest classification accuracy under out-of-bag estimation, repeated stratified cross-validation (mean ± SD), and leave-one-site-out validation, in which all replicates from a collection site, or from a sampling region for the organ analysis, were held out together; dashed horizontal lines indicate chance levels. (**D**) PCA of tuberous-root samples coloured by macro-geographic zone (subset S2); dotted reference lines indicate zero on the PC1 and PC2 axes.

For the 51 tuberous-root samples from 17 sites in seven provinces, PCA showed only modest separation, the first three components explaining 24.2%, 14.5% and 11.2% of the variance (about 50% cumulatively) and no clear provincial clustering (Figure 4); grouping the same samples by macro-geographic zone did not improve the separation (Figure 5D). Under supervision, random forest nevertheless separated the samples effectively: out-of-bag accuracies were 88.2% for the 17 sites, and 90.2% for the seven provinces, and PERMANOVA confirmed a significant site effect (pseudo-F = 7.8, *p* = 0.001; Table 2). These figures, however, quantify separability within the present sample set rather than the ability to classify an independently collected sample, because the three replicates of each site were prepared from the same bulked field material and are therefore not independent. Repeated stratified cross-validation, which shares replicates in the same way, gave similar figures (province 84.9 ± 11.8%; macro-geographic zone 86.3 ± 7.6%; mean ± SD over 5 repeats). When the validation was tightened so that all three replicates of a site were held out together, accuracy fell from 88.9% to 42.2% for province (five provinces represented by at least two sites; chance level 20%) and from 88.2% to 52.9% for macro-geographic zone (chance level 25%), whereas the equivalent test on the organ data set, in which an entire sampling region was held out, left organ classification unchanged at 100% (Figure 5C). Permutation tests confirmed that all three classifications performed far better than chance (*p* = 0.01, 99 permutations), so the geographic signal is real; but it is largely specific to the individual site and, at the level of transferable structure, only the coarse macro-geographic zone retained an accuracy about twice the chance level.

### 3.7. Robustness of the Conclusions to Annotation Stringency

The effect of annotation filtering on PCA was dataset-dependent (Figure 5A; Table 3). In the origin data set, PC1 increased from 26.6% for all annotations to 47.8% for the flavonoid-and-phenolic-acid subset, showing that feature filtering substantially changed the unsupervised variance structure. In the organ data set, the change was smaller, from 24.8% to a maximum of 30.1%.

PERMANOVA effect sizes were comparatively stable across the same subsets (Figure 5B; Table 3). Pseudo-F ranged from 16.4 to 17.9 for organ and from 3.5 to 4.2 for sampling region, while random-forest organ classification remained between 97.5% and 100%. These results describe robustness of the organ-to-region effect-size contrast; they do not compensate for incomplete annotation of individual metabolites.

Direct RAW-file inspection provided an extracted-ion chromatogram and a matched negative-mode product-ion spectrum for the feature putatively annotated as isoorientin 2”-O-gallate (Figure S3). The observed DDA precursor at *m*/*z* 599.1041 had a mass error of −0.30 ppm relative to the theoretical [M-H]- value of *m*/*z* 599.1042. Product ions at *m*/*z* 447.094, 357.059 and 327.052 were consistent with loss of a galloyl moiety and subsequent flavone C-glycoside cross-ring fragmentation. The kaempferol-series audit covered 306 RAW files (153 in each ionisation mode) and six queried isobaric formulas per mode. None of the 1836 file-target combinations had a DDA precursor match within ±5 ppm. Exact-mass chromatographic signals were widespread, but their retention times varied, and signals at some masses also occurred in solvent blanks. An expanded product-ion search retrieved 1357 spectra containing a kaempferol/luteolin-type aglycone ion at the defined threshold, but none combined this ion with a neutral loss matching the common queried glycosides within 20 ppm. No compound in the kaempferol series was therefore assigned, and compound-specific abundance was not estimated.

## 4. Discussion

This study identified a strong organ-specific metabolomic pattern and a weaker, largely site-specific geographic pattern in *T. hemsleyanum*. The organ result was supported by both the chemical composition of the three tissues and the multivariate analyses.

The tuberous root had the largest shares of amino acids and derivatives (58%) and lipids and fatty acids (52%). These pools are consistent with reserve storage and membrane turnover in a below-ground storage organ. The stem had the largest shares of soluble sugars and glycosides (56%) and phenolic acids (48%), consistent with transport and supporting functions. The leaf had the largest shares of flavonoids (51%) and compounds annotated as alkaloids (44%), which is consistent with the protective roles of secondary metabolites in photosynthetic tissue [8,12,14,19].

Hierarchical clustering supported these class-level patterns. Leaves were relatively enriched in putative 4,5-dicaffeoylquinic acid and in flavone C-glycoside conjugates of the isovitexin and isoorientin series. The predominance of glycosides in leaves is biologically plausible because glycosylation increases solubility and supports vacuolar storage [11,13]. Tuberous roots were relatively enriched in several nitrogen-containing compounds, phenolic acids and lipids. These individual annotations remain putative and are used as supporting evidence only.

The qualitative organ differences were consistent with the quantitative analyses. PCA separated roots, stems and leaves, and random-forest classification reached 100% out-of-bag accuracy. PERMANOVA gave a pseudo-F of 16.7 for organ and 4.1 for sampling region within the organ data set. Organ classification also remained 100% when an entire sampling region was withheld. Together, these results show that the organ pattern generalised across the sampled regions.

The phenolic component broadly agrees with published descriptions of *T. hemsleyanum*. Dicaffeoylquinic acids, flavone C-glycosides of the vitexin and orientin series, and quercetin glycosides have been reported previously [1,2,3,22]. Kaempferol and its glycosides, including kaempferol-3-O-rutinoside, were not definitively identified in the present data set [6].

The RAW-file audit queried six kaempferol-series isobaric formulas in both ionisation modes across every study sample, QC file and solvent blank. Exact-mass signals were widespread, but their retention times varied, and signals at some masses were also observed in blanks. None had a matching DDA precursor within ±5 ppm. An expanded search found non-specific spectra containing a kaempferol/luteolin-type aglycone ion, but no diagnostic neutral loss connected these spectra to the common queried glycosides. These results do not demonstrate a true biological absence. They show that the available untargeted data cannot distinguish kaempferol from luteolin or assign their isomeric glycosides. Compound-specific relative abundance could not be calculated.

The final Compound Discoverer table did not contain features assigned to these canonical kaempferol compounds. Because the complete intermediate screening history was not preserved, the specific processing step responsible for their exclusion cannot be determined. The short untargeted gradient and Top-6 DDA scheme reduced the opportunity to fragment co-eluting isobars. Targeted LC-MS/MS with authentic standards would be required to establish their identities and concentrations.

Feature filtering affected the two PCA analyses differently. In the origin data set, PC1 increased from 26.6% to 47.8% after restriction to flavonoids and phenolic acids. This substantial increase shows that primary and unassigned features influenced the unsupervised variance structure of the origin data. The corresponding organ PC1 change was smaller, from 24.8% to a maximum of 30.1%. PERMANOVA pseudo-F nevertheless remained between 16.4 and 17.9 for organ and between 3.5 and 4.2 for sampling region across the five subsets. These observations address different properties of the data and do not compensate for incomplete annotation of individual metabolites.

The geographic results require more cautious interpretation. Out-of-bag and repeated cross-validation accuracy was 84.9–90.2% when replicates from the same collection site could occur in both training and test sets. When all replicates from one site were withheld together, accuracy fell to 42.2% for province and 52.9% for macro-geographic zone. The geographic signal was therefore real but largely site-specific. Soil, shading, plant age, microhabitat and genotype may all contribute. The present sampling design cannot support an origin-authentication system [23,24,26].

The organ pattern has a practical implication. Leaves carried the largest relative share of flavonoids and many organ-enriched metabolites, which supports further evaluation of the aerial parts as a supplementary resource. Targeted quantification against authentic standards and biological activity assays are still required before pharmacological value can be inferred.

Several limitations remain. Most annotations were MSI Level 3, and no targeted MS/MS experiment or authentic standard was available to confirm the kaempferol-series compounds. The RAW-file audit could not resolve the isomeric assignments, and the intermediate Compound Discoverer exclusion history was unavailable. The sampled plants were documented photographically, but a formal voucher specimen was not retained. The organ and origin comparisons were analysed in different batches. In addition, three replicates from one bulked collection describe a site but do not represent independent regional sampling. All material was collected in one season, so seasonal and inter-annual variation remains to be characterised.

## 5. Conclusions

Using UHPLC-Orbitrap MS-based untargeted metabolomics with MSI confidence grading and explicit annotation curation, this study characterised the *T. hemsleyanum* metabolome along an organ and a geographic dimension. Organ identity is the dominant factor: root, stem and leaf metabolomes are completely separable (random forest out-of-bag accuracy 100%), the organ effect is about four times that of sampling region, the separation is unaffected when an entire sampling region is withheld from the model, and each organ shows a class specialisation that matches its physiological role, with the tuberous root carrying the largest share of the amino-acid and lipid pools, the stem of the soluble sugars and phenolic acids, and the leaf of the flavonoids. These patterns proved insensitive to the stringency of annotation curation and can therefore be regarded as properties of the plant rather than of the data-processing workflow. Geographic differences among tuberous roots, by contrast, are significant but largely site-specific: supervised accuracy falls from about 89% to 42% once an entire, previously unseen collection site is held out, and only a coarse climatic grouping retains modest transferable structure. The metabolome of *T. hemsleyanum* thus provides a firm basis for reasoning about which organ to use, and for regarding the aerial parts as an under-used resource, but does not, on the present evidence, provide a basis for origin authentication.

## Figures and Tables

**Table 1:** Sampling information for the organ and origin sample sets of *T. hemsleyanum*.

Set	Code	Region	Province	Plant Part
Organ	FJ-NP	Nanping	Fujian	Root/Stem/Leaf
Organ	ZJ-HZ	Hangzhou	Zhejiang	Root/Stem/Leaf
Organ	ZJ-JH	Jinhua	Zhejiang	Root/Stem/Leaf
Organ	GX-BS	Baise	Guangxi	Root/Stem/Leaf
Organ	HB-NS	Enshi	Hubei	Root/Stem/Leaf
Organ	HN-YAO	Yongzhou (S)	Hunan	Root/Stem/Leaf
Organ	HN-YONG	Yongzhou (N)	Hunan	Root/Stem/Leaf
Organ	JX-GZ	Ganzhou	Jiangxi	Root/Stem/Leaf
Organ	SC-WY	Wanyuan	Sichuan	Root/Stem/Leaf
Origin	ZJ-WZ	Wenzhou	Zhejiang	Tuberous root
Origin	ZJ-JH	Jinhua	Zhejiang	Tuberous root
Origin	ZJ-NB	Ningbo	Zhejiang	Tuberous root
Origin	ZJ-TZ	Taizhou	Zhejiang	Tuberous root
Origin	ZJ-LS	Lishui	Zhejiang	Tuberous root
Origin	FJ-FZ	Fuzhou	Fujian	Tuberous root
Origin	FJ-ND	Ningde	Fujian	Tuberous root
Origin	FJ-SM	Sanming	Fujian	Tuberous root
Origin	GZ-QXN	Qianxinan	Guizhou	Tuberous root
Origin	GZ-QDN	Qiandongnan	Guizhou	Tuberous root
Origin	JX-ZS	Zhangshu	Jiangxi	Tuberous root
Origin	GD-QY	Qingyuan	Guangdong	Tuberous root
Origin	GX-LZ	Liuzhou	Guangxi	Tuberous root
Origin	GX-YL	Yulin	Guangxi	Tuberous root
Origin	HN-HH	Huaihua	Hunan	Tuberous root
Origin	HN-YZ	Yongzhou	Hunan	Tuberous root
Origin	HN-SY	Shaoyang	Hunan	Tuberous root

Each organ/part and each origin had three biological replicates (organ set: 9 regions × 3 organs × 3 = 81 samples; origin set: 17 origins × 3 = 51 samples). All samples were obtained from cultivated plants.

**Table 4:** Identification evidence for representative annotated compounds, and outcome of a targeted search for the canonical flavonoids of *T. hemsleyanum*.

Putative Compound	Formula	Mode	Measured *m*/*z*	Error (ppm)	RT (min)	MS/MS	MSI Level
4,5-Dicaffeoylquinic acid	C_25_H_24_O_12_	ESI^−^	515.11980	0.6	5.24	Yes	3
Isovitexin 2″-O-(6‴-(E)-p-coumaroyl)glucoside 4′-O-glucoside	C_42_H_46_O_22_	ESI^−^	901.24099	0.2	4.64	Yes	3
Isovitexin 2″-O-(6‴-(E)-feruloyl)glucoside 4′-O-glucoside	C_43_H_48_O_23_	ESI^−^	931.25145	0.1	4.64	Yes	3
Vitexin 4′-O-glucoside-2″-O-rhamnoside	C_33_H_40_O_19_	ESI^−^	739.20889	−0.3	3.39	Yes	3
Isoorientin 2″-O-gallate	C_28_H_24_O_15_	ESI^−^	599.10472	0.8	6.46	Yes	3
Apigenin 7-(6‴-acetylallosyl-(1→2)glucoside)	C_29_H_32_O_16_	ESI^−^	635.16179	0.1	2.79	Yes	3
Myricetin 3-sambubioside	C_26_H_28_O_17_	ESI^−^	611.12554	0.3	5.57	Yes	3
Quercetin 3-(2″-galloyl-alpha-L-arabinopyranoside)	C_27_H_22_O_15_	ESI^−^	585.08770	−1.5	0.61	Yes	3
Kaempferol	C_15_H_10_O_6_	ESI^−^	not retained	-	-	-	-
Kaempferol-3-O-rutinoside	C_27_H_30_O_15_	ESI^−^	not retained	-	-	-	-
Kaempferol-3-O-glucoside	C_21_H_20_O_11_	ESI^−^	not retained	-	-	-	-
Kaempferol-3-O-rhamnoside	C_21_H_20_O_10_	ESI^−^	not retained	-	-	-	-
Quercetin	C_15_H_10_O_7_	ESI^−^	not retained	-	-	-	-
Rutin	C_27_H_30_O_16_	ESI^−^	not retained	-	-	-	-
Chlorogenic acid	C_16_H_18_O_9_	ESI^−^	not retained	-	-	-	-

Table note: The eight compounds in the upper part of the table are representative annotations retained after curation. The seven compounds in the lower part are canonical constituents of *T. hemsleyanum* for which the complete 615-compound Compound Discoverer output was searched by exact neutral mass (±5 ppm), irrespective of assigned name. “Not retained” means that no corresponding feature was present in that final output. The complete intermediate Compound Discoverer screening history was not preserved, so exclusion cannot be attributed to a specific processing or frequency-filtering step. A separate audit queried six kaempferol-series isobaric formulas in all 132 study samples, 15 QC files and six solvent blanks in each ionisation mode. No queried mass had a matching DDA precursor within ±5 ppm. Exact-mass signals alone were non-specific because retention times varied and some signals also occurred in blanks. No kaempferol-series assignment or compound-specific abundance estimate was therefore made. All retained annotations shown here are MSI Level 3 (putative). Figure S3 provides the raw EIC and matched product-ion spectrum for putative isoorientin 2″-O-gallate.

## Data Availability

The original contributions presented in the study are included in the article and its Supplementary Materials. The curated feature table, the definitions of the five annotation subsets used in the sensitivity analysis and the scripts used to generate every statistic and figure reported here are available from the corresponding author on request, so that all reported values can be regenerated from the Compound Discoverer output; further inquiries can be directed to the corresponding authors.

## Supplementary Materials

The following supporting information can be downloaded at: https://www.mdpi.com/article/10.3390/metabo16090619/s1, Figure S1: Quantification of the contributions of organ identity and sampling region to metabolic variation. (A) PERMANOVA effect sizes (pseudo-F, with R^2^ and P annotated); (B) confusion matrix of the random-forest organ classification (out-of-bag accuracy = 100%). The numerical values are reported in Table 2 of the main text; Figure S2: Differential metabolites among organs. (A–C) Volcano plots for the root–stem, root–leaf and stem–leaf comparisons; (D) Venn diagram of the organ-enriched metabolites. Screening criteria were |log2 fold change| > 1 and P < 0.05; Figure S3: Raw-file chromatographic and product-ion evidence for the feature putatively annotated as isoorientin 2″-O-gallate (MSI Level 3) in negative ion mode. (A) Extracted-ion chromatogram at m/z 599.1047 ± 5 ppm in tuberous-root sample 1-2 (Wenzhou). The Compound Discoverer feature eluted at 6.46 min; the DDA event at 6.36 min is marked by the dashed line and corresponds to the earlier peak (apex, 6.43 min). The stronger later peak at 6.79 min is an isobaric signal and is not assigned. (B) Product-ion spectrum acquired from precursor m/z 599.1041. The theoretical [M−H]^−^ value for C28H24O15 is m/z 599.1042, giving a mass error of −0.30 ppm. Product ions at m/z 447.094, 357.059 and 327.052 are consistent with loss of a galloyl moiety and subsequent flavone C-glycoside cross-ring fragmentation. No authentic standard was analysed; the annotation therefore remains putative; Figure S4: Representative *Tetrastigma hemsleyanum* material photographed during sample collection. (A) Complete climbing plant showing the aerial vine and swollen underground organs; (B) leaves; (C) stems; and (D) tuberous roots, including cut surfaces. The photograph documents the field morphology of the sampled material. A formal voucher specimen was not retained. The original Chinese labels were masked during figure preparation; no plant material in the image was altered.
