# Supplementary material for: Organ Identity Outweighs Geographic Origin in Shaping the Metabolome of Tetrastigma hemsleyanum
Source: Metabolites. 2026 Aug 27;16(9):619. doi: 10.3390/metabo16090619 (PMC13608988; doi:10.3390/metabo16090619)
Supplement: Supplementary file 1 [file metabolites-16-00619-s001.zip › metabolites-4460053-supplementary.pdf]

Supplementary Materials

Organ identity outweighs geographic origin in shaping the metabolome of Tetrastigma hemsleyanum

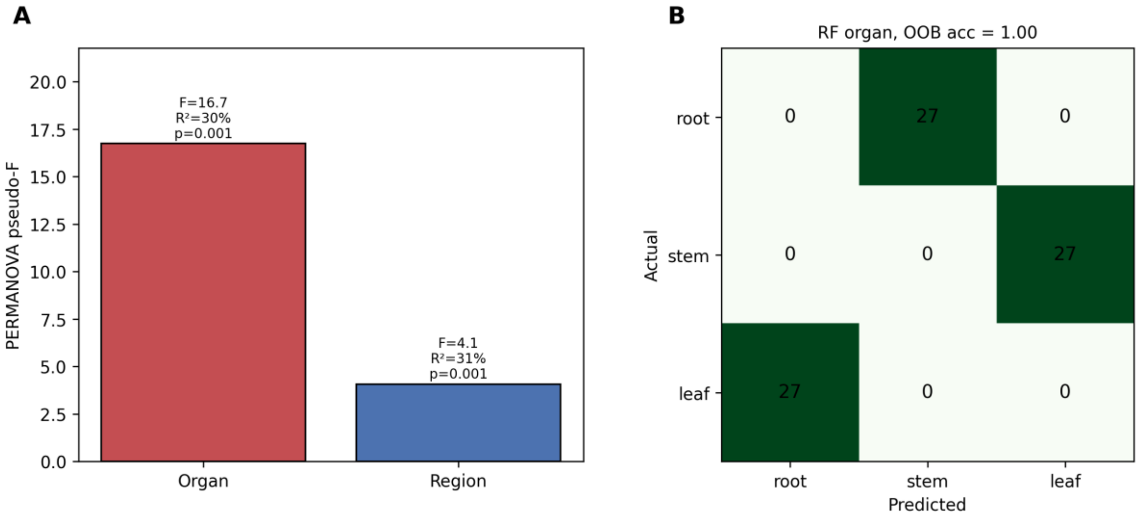

Figure S1. Quantification of the contributions of organ identity and sampling region to metabolic variation. (A) PERMANOVA effect sizes (pseudo-F, with R^2 and P annotated); (B) confusion matrix of the random-forest organ classification (out-of-bag accuracy = 100%). The numerical values are reported in Table 2 of the main text.

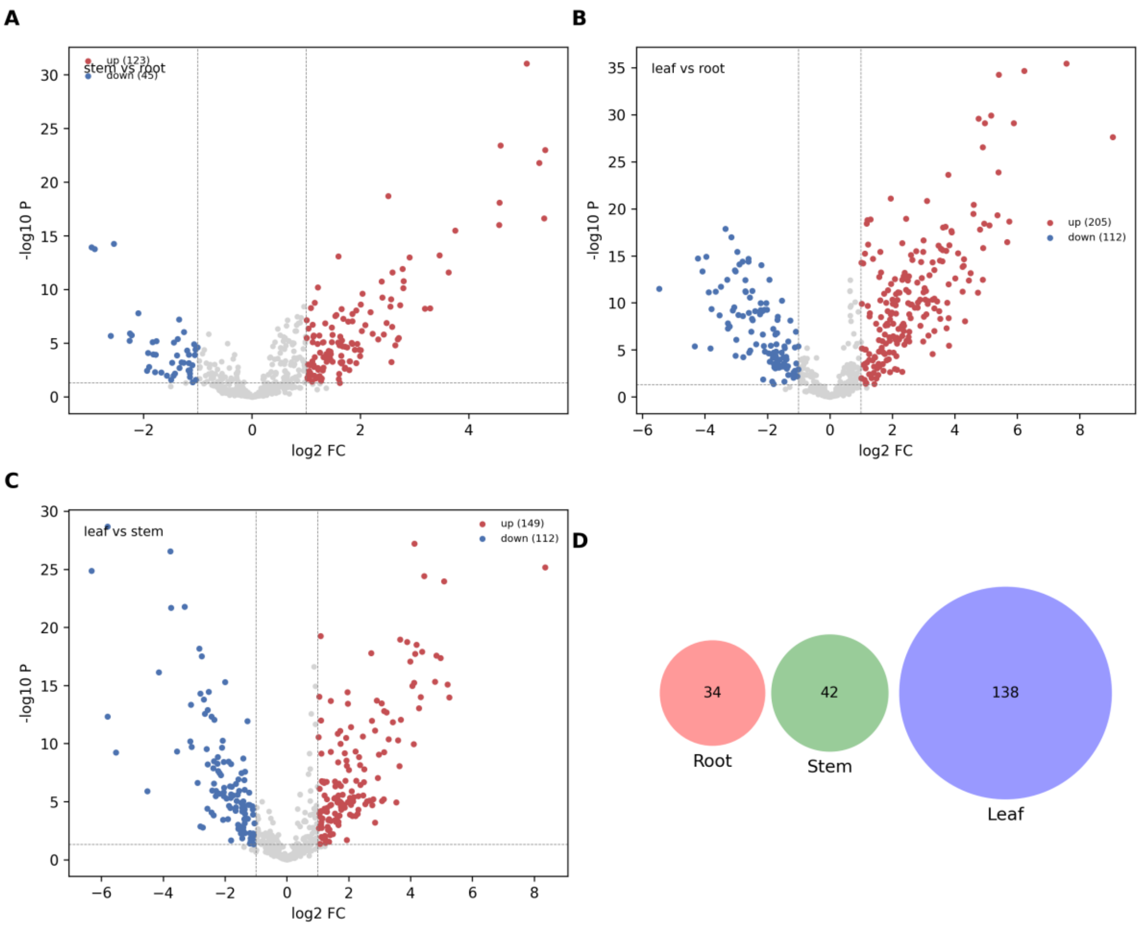

Figure S2. Differential metabolites among organs. (A–C) Volcano plots for the root–stem, root–leaf and stem–leaf comparisons; (D) Venn diagram of the organ-enriched metabolites. Screening criteria were |log2 fold change| > 1 and P < 0.05.

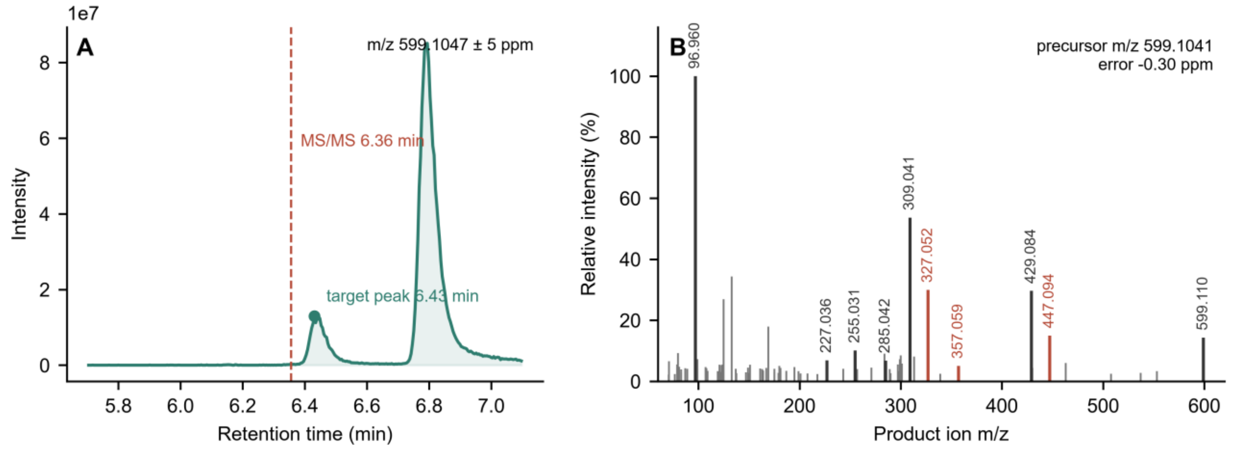

Figure S3. Raw-file chromatographic and product-ion evidence for the feature putatively annotated as isoorientin 2"-O-gallate (MSI Level 3) in negative ion mode. (A) Extracted-ion chromatogram at m/z 599.1047 ± 5 ppm in tuberous-root sample 1-2 (Wenzhou). The Compound Discoverer feature eluted at 6.46 min; the DDA event at 6.36 min is marked by the dashed line and corresponds to the earlier peak (apex, 6.43 min). The stronger later peak at 6.79 min is an isobaric signal and is not assigned. (B) Product-ion spectrum acquired from precursor m/z 599.1041. The theoretical [M-H]- value for $C_{28}H_{24}O_{15}$ is m/z 599.1042, giving a mass error of -0.30 ppm. Product ions at m/z 447.094, 357.059 and 327.052 are consistent with loss of a galloyl moiety and subsequent flavone C-glycoside cross-ring fragmentation. No authentic standard was analysed; the annotation therefore remains putative.

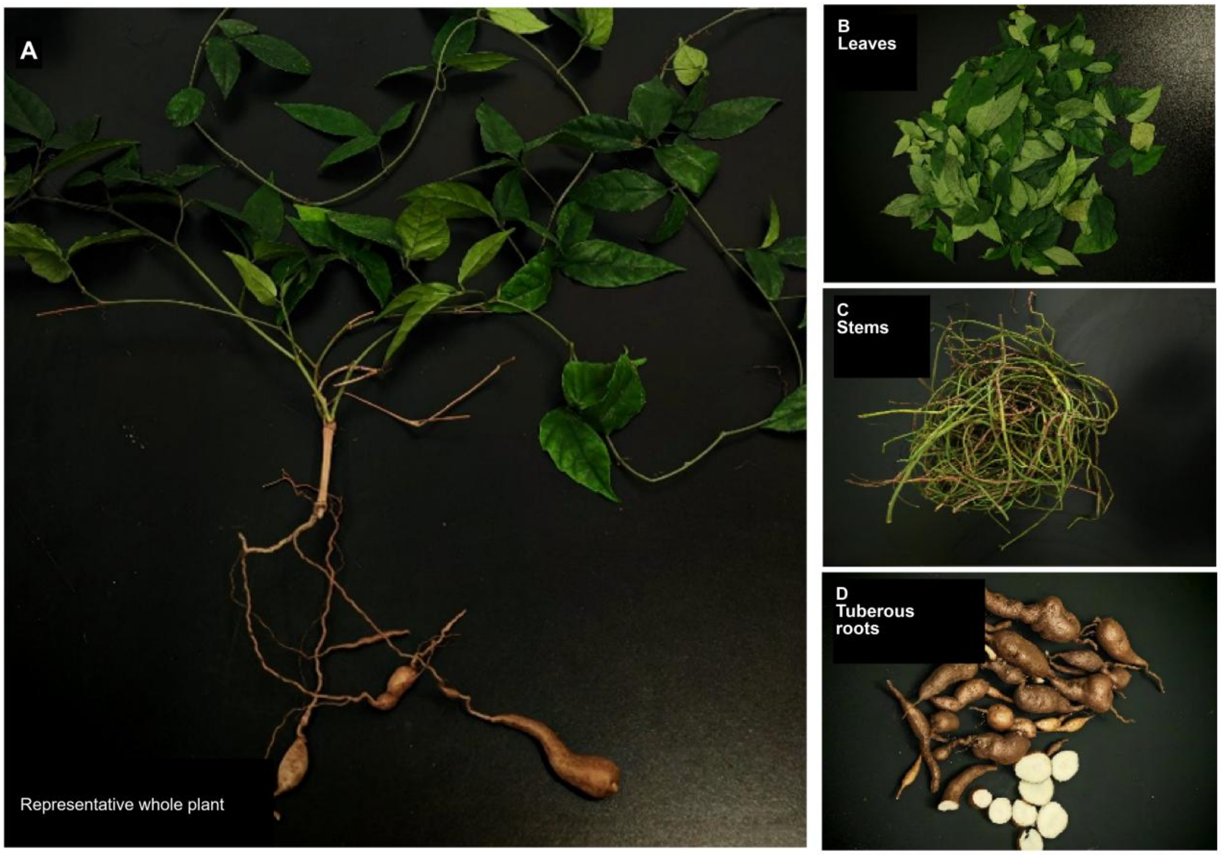

Figure S4. Representative *Tetrastigma hemsleyanum* material photographed during sample collection. (A) Complete climbing plant showing the aerial vine and swollen underground organs; (B) leaves; (C) stems; and (D) tuberous roots, including cut surfaces. The photograph documents the field morphology of the sampled material. A formal voucher specimen was not retained. The original Chinese labels were masked during figure preparation; no plant material in the image was altered.
